# Progress of Sanitary Legislation

**Published:** 1855-06

**Authors:** 


### HYGIENIC JURISPRUDENCE.

### PROGRESS OF SANITARY LEGISLATION.

### PARLIAMENTARY BILLS AND ACTS.

*The Public Health Bill* and *Nuisances Removal and Diseases Prevention Bill,* as amended by the Select Committee, have been brought out during the past quarter. The Public Health Bill legislates only for England.* This act provides, 1. That a Board of Health be constituted, consisting of a president, the principal secretaries of state, and the president and vice-president for the time being of the committee of council appointed for the consideration of matters relating to trade and foreign plantations. 2. That the General Board may appoint a medical council. 3. That sanitary inspectors may be sent from the General Board to any locality. 4. That local boards of health be formed, consisting in boroughs, of the mayor, aldermen, and burgesses; in other districts, of either the existing local boards, or boards of improvement commissioners elected by the rate-payers, or, under some circumstances, of such elective members as may be determined by a resolution of the rate-payers. Such local boards to be vested with considerable power in regard to nuisances, sewerage, buildings, scavenging, offensive trades, running streams, receiving refuse, roads, supply of water, lighting, slaughter-houses, and pleasure grounds. These boards are also to have the power of appointing an officer of health, and inspector of nuisances.

*The Nuisances Removal and Diseases Prevention Acts Consolidation and Amendment Bill,* provides that the word "Nuisances" include any premises, or ditch, gutter, water-course, privy, urinal, cesspool, drain, or ashpit, so foul as to be offensive or injurious to health; any animal so kept as to be injurious to health; or any accumulation or deposit within the limits of any town or street, or less than fifty yards from any house, offensive or injurious to health.

Under this act powers also are given to local authorities to inspect, prevent, or remove nuisances. The penalties against all nuisances are summary and sufficiently severe. Considering these two acts together, we cannot but hope that they will become law. It is a pity, however, that they should be disunited; since as regards the great principles involved, the objects of both are nearly identical. It is impossible also to conceal that the construction of the General Board of Health, as proposed in the Health Bill, is faulty. Are the duties of the principal secretaries of state, and of the president and vice-presidents of the Committee of Council on Trade and Foreign Plantations, so trifling, that these gentlemen can spare time to consider, as they deserve, the

* London alone is excepted.

important questions of sanitary and social science? We presume not. Neither, in plain language, but with all respect, do we think that these important functionaries, as they are now selected, are the men fitted for this peculiar duty. Are they men of science? Are they chemists, mechanists, engineers, physiologists, physicians? Do they, from personal experience, understand anything relating to the sanitary wants of the community? On the contrary, many of these qualifications would flatly disqualify a man from becoming secretary of state. The presidency of the board too is left uncertain. So long as it remains in the hands of Sir Benjamin Hall, it is well represented; since he has, with praiseworthy diligence, made himself master of the practical workings of sanitary legislation. But, as the office may change, what do we know about succeeding presidents? or that the proper qualifications for that office will be considered?

If ever a Board of Health is to hold its true position in this country, it must consist of men, under the presidency of a competent minister, who are acquainted with the principles of sanitary administration, and with the general laws of sanitary science. They must not merely have a vague idea of the causes of disease, but must know, too, the nature of disease itself. We shall receive little benefit from sanitary legislation until these broad and common sense views are accepted and acted upon.

*Dwelling-houses for the Poor in Scotland and Ireland.*—Two bills have been introduced in the House of Commons regarding the provision of improved dwellings for the working classes. One applies to Ireland—the other to Scotland. The first was brought in by Sir W. Somerville and Mr. George Hamilton—the second by Mr. Dunlop, Mr. Baird, and Mr. Cowan. The objects of both bills are excellent.

*Burial Grounds in Scotland.*—A bill to amend the laws concerning the burial grounds of the dead in Scotland also deserves attention. It is prepared by the Lord Advocate and Sir George Grey. The bill provides for burial grounds free from danger to health; for opening burial grounds; for their management under burial boards; and for places for the reception of bodies previous to interment.

### SEWERS.—HOUSE DRAINAGE.

*The House Drainage Act,* which has long been before Parliament, has received the Royal assent. It enables the Metropolitan Commissioners to carry on works of improvements, house drainage, and cleansing (to the extent of an annual cost of £2,500) either on separate or individual tenements, the cost to be charged to the owners or occupiers.

### SANITARY REPORTS AND RETURNS.

*Quarantine; Sanitary state of Gibraltar.*—Two returns—one on Quarantine in Gibraltar, the other on the sanitary state of that island—have been made for the Government by Dr. Baly. In reference to Quarantine, Dr. Baly remarks that persons arriving at Gibraltar from England during the existence of Quarantine, should no longer be subjected to the discomfort of detention in hulks, but should be accommodated in a lazaretto provided for their reception. The second return of the sanitary condition of Gibraltar is a short, but interesting, document. The sewerage is defective; the effluvia from drains offensive; and the supply ofwater scanty.

*A Report on the Treatment of Cholera* has been drawn up by the Medical Council of the Board of Health. Many plans of treatment are detailed, with the usual success of bringing out the painful fact that there is as yet no treatment for cholera. *Apropos* to this, the London homœopaths have sent in anything but an infinitesimal dose of wrath, because the Council have not chosen to notice their absurdities. In a return to the House they publish a list of cases and *cures*, which show only two things; first, that homœopaths give enormous doses of camphor (at the rate of four grains an hour); and secondly, that cases of cholera may get well without any treatment of a physical kind, which everybody knew before.

*Dr. Sutherland's Report on Cholera in London in* 1854 is an interesting document, showing mainly the old fact that uncleanliness and disease go hand in hand.

*Returns relating to Burial Grounds, Metropolis.*—Out of 85 of these grounds 11 are to be closed within the next four years.

*Water Companies.*—The Southwark and Vauxhall Water Company have written to the Government stating that their new works, which will enable them to get water from Hampton, are nearly completed. The Hampstead Company have also nearly finished their works; source of supply an artesian well. The East London Company's works are equally progressing; source of supply the river Lea in Essex. The Chelsea Company report progress; supply Seething wells, above Kingston-on-Thames. The Kent Waterworks have complied with the provisions of the act of 1852. The New River Company are continuing their work satisfactorily, and also the Grand Junction Waterworks Company, which derives its supply from Hampton. The West Middlesex Waterworks progress; source of supply above Hampton. The Lambeth Waterworks will be completed as soon as a reservoir has been covered. These Companies deserve public commendation for their labours. In spite of enormous difficulties, and at immense cost, they are doing their best to secure the great want of London—pure water.

*The Metropolitan Commission of Sewers* has presented an elaborate abstract of its contracts and works completed during the year.

*Vaccination.*—The Annual Report of the National Vaccine Board shows that 9,198 persons have been reported as vaccinated by the stationary vaccinators, and 153,510 by other practitioners; that vaccine lymph has been abundantly sent to different parts of the world; that vaccination is still imperfectly performed in some parts of England; and that compulsory vaccination is most necessary.

*Health of Wigston Magna, Leicestershire.*—A report by Mr. A. Dickens indicates how much disease depends on the absence of sanitary measures. This little country village, from mere neglect, has an average mortality as high as London itself, viz., 33 in 1,000. Typhus fever is the main cause of this mortality.

### LETTERS.

*Smoke Nuisance Abatement.*—An interesting letter on consumption of smoke has been submitted to Lord Palmerston by the Secretary of the Board of Health. The letter shows that the emission of smoke is a great waste of fuel; that the impediment to the prevention of smoke in manufactories is the insufficient boiler surface in proportion to the steam required; that the reduction of smoke to a small amount may easily be effected; and that the makers of furnaces are guided by mere empirical rules instead of acting on scientific principles the result of experiment.

We may add that the Prefect of the Seine issued, on November 11th, 1854, a stringent *ordonnance* for the complete prevention of smoke in Paris in the course of six months. The Prefect argues in the preamble, that the prevention of smoke is easy, and that the proprietors of workshops are obliged by the terms of their licence to submit to such conditions necessary for the preservation of public health as the Government may think fit to enforce.

*Preparations for Cholera.*—In a letter, dated May 12th, Sir B. Hall directs the attention of local authorities to the principal laws relating to nuisances and other causes of disease, especially cholera.

### ADDITIONAL NOTES.

*Adulteration of Food.*—On the motion of Mr. Scholefield, a Parliamentary committee has been appointed to inquire into the adulteration of food, drinks, and drugs.

*Extramural Interments.*—An important discussion recently took place in the House of Peers on this matter, in which the Bishop of London, Lord Shaftesbury, and Lord Redesdale joined. The sum of the discussion is given in the first article of this number of The Journal of Public Health.

*Public Health Bill.*—Several petitions have been sent in respecting this Bill: one is from the College of Physicians, recommending that medical supervision of districts should not only take place when an epidemic is present, but at all other times.

A large book has been compiled out of the evidence taken by the framers of the new Public Health Bill. The two chief features of the work are, Dr. Farr's admirable statistical facts, and Dr. Snow's extreme assertion that foul smells are not absolutely productive of disease.

*Employment of Females and young Persons in Factories, &c.*—Mr. Butt has obtained leave to bring in a Bill to regulate the employment of females and young persons under eighteen in bleaching, finishing, and dyeing works.

